# Quantitative proteomic analysis of host—pathogen interactions: a study of *Acinetobacter baumannii* responses to host airways

**DOI:** 10.1186/s12864-015-1608-z

**Published:** 2015-05-30

**Authors:** Jose Antonio Méndez, Jesús Mateos, Alejandro Beceiro, María Lopez, María Tomás, Margarita Poza, Germán Bou

**Affiliations:** Microbiology Division, INIBIC-Complejo Hospitalario Universitario de la Coruña, A Coruña, Spain; Grupo de Proteomica-PBR2-ProteoRed/ISCIII-Servicio de Reumatologia, A Coruña, Spain

**Keywords:** Proteome, *Acinetobacter baumannii*, Host-pathogen interaction, *Ex vivo*, Virulence

## Abstract

**Background:**

*Acinetobacter baumannii* is a major health problem. The most common infection caused by *A. baumannii* is hospital acquired pneumonia, and the associated mortality rate is approximately 50 %. Neither *in vivo* nor *ex vivo* expression profiling has been performed at the proteomic or transcriptomic level for pneumonia caused by *A. baumannii*. In this study, we characterized the proteome of *A. baumannii* under conditions that simulate those found in the airways, to gain some insight into how *A. baumannii* adapts to the host and to improve knowledge about the pathogenesis and virulence of this bacterium. A clinical strain of *A. baumannii* was grown under different conditions: in the presence of bronchoalveolar lavage fluid from infected rats, of RAW 264.7 cells to simulate conditions in the respiratory tract and in control conditions. We used iTRAQ labelling and LC-MALDI-TOF/TOF to investigate how *A. baumannii* responds on exposure to macrophages/BALF.

**Results:**

179 proteins showed differential expression. In both models, proteins involved in the following processes were over-expressed: (i) pathogenesis and virulence (OmpA, YjjK); (ii) cell wall/membrane/envelope biogenesis (MurC); (iii) energy production and conversion (acetyl-CoA hydrolase); and (iv) translation (50S ribosomal protein L9). Proteins involved in the following were under-expressed: (i) lipid metabolism (short-chain dehydrogenase); (ii) amino acid metabolism and transport (aspartate aminotransferase); (iii) unknown function (DNA-binding protein); and (iv) inorganic ion transport and metabolism (hydroperoxidase).

**Conclusions:**

We observed alterations in cell wall synthesis and identified 2 upregulated virulence-associated proteins with >15 peptides/protein in both *ex vivo* models (OmpA and YjjK), suggesting that these proteins are fundamental for pathogenesis and virulence in the airways. This study is the first comprehensive overview of the *ex vivo* proteome of *A. baumannii* and is an important step towards identification of diagnostic biomarkers, novel drug targets and potential vaccine candidates in the fight against pneumonia caused by *A. baumannii*.

**Electronic supplementary material:**

The online version of this article (doi:10.1186/s12864-015-1608-z) contains supplementary material, which is available to authorized users.

## Background

The World Health Organization has recently identified antimicrobial resistance as one of the three most important problems facing human health. The most common and serious multidrug-resistant pathogens have been encompassed within the acronym “ESKAPE”, which stands for *Enterococcus faecium*, *Staphylococcus aureus*, *Klebsiella pneumoniae*, *Acinetobacter baumannii, Pseudomonas aeruginosa* and *Enterobacter* spp. [1]. *Acinetobacter baumannii* is an important opportunistic nosocomial pathogen that is often associated with epidemic outbreaks of infection. This organism is frequently pandrug-resistant and is capable of causing substantial morbidity and mortality in patients with severe underlying disease, both in the hospital and in the community [2]. *Acinetobacter baumannii* is an uncommon but important cause of community-acquired pneumonia, which appears to be a unique clinical entity occurring predominantly in tropical climates. This community-acquired pneumonia appears to be characterized by a fulminant course, with acute onset of dyspnea, cough and fever followed by rapid progression to respiratory failure and shock. The mortality rate is high (40–64 %) [3, 4]. Nosocomial pneumonia is the most important infection caused by *A. baumannii* and is particularly associated with the application of mechanical ventilatory procedures [5]. The crude mortality rate associated with nosocomial Acinetobacter infections has been reported to range from 20 to 45 % [6, 7]. A prospective study of 240 *A. baumannii* infections showed that > 90 % of infections were nosocomially acquired and that only 4 % were community acquired; moreover, respiratory track infections were the most common (39.3 %) [8]. *Acinetobacter baumannii* has a propensity to cause outbreaks, probably because of its ability to survive desiccation and its multidrug resistance, amongst other reasons [9]. Although *A. baumannii* is only rarely isolated from soil, vegetables, animals, humans and inanimate surfaces that are often in contact with humans [10], the natural habitats of *A. baumannii* remain to be established [11]. In the community, *A. baumannii* is a rare colonizer of human skin in temperate climates, although skin carriage is more common in tropical environments [12, 13]. In Australia, wet-season throat carriage of *A. baumannii* was found in 10 % of community residents with excess levels of alcohol consumption [3]. The bacterium is ubiquitous in the hospital setting (*e.g.*, bedside, bedrail, ventilator, infusion pump, pillow, resuscitation equipment, washbasins) [14, 15]. Furthermore, *A. baumannii* is found to persist as a contaminant of the hands, gloves and gowns of healthcare workers [16, 17]. This bacterium can survive on inanimate objects for long periods, even after exposure to dry conditions [18] and is also capable of resisting physical and chemical disinfection, often by forming a biofilm [19]. It has a remarkable ability to up-regulate or acquire resistance determinants, making it one of the most important microorganisms threatening the current antibiotic era [11]. Numerous outbreaks of pandrug-resistant *A. baumannii* have been documented in Asian and Middle East hospitals. Resistance to both tigecycline and polymyxin B (drugs relied on heavily to treat infection with *A. baumannii*) already exists in these regions [20, 21].

The first genome of an *Acinetobacter* spp. to be sequenced was that of the highly transformable *Acinetobacter* spp. strain ADP1 in 2004 [22]. The first completed *A. baumannii* genome was reported for strain ATCC 17978 in 2007 and included a considerable number of the island-containing genes (16) implicated in virulence, indicating that several of the microorganism’s genes are devoted to pathogenesis. The largest island contains elements homologous to the *Legionella*/*Coxiella* Type IV secretion apparatus. Type IV secretion systems have been demonstrated to be important for virulence in other microorganisms and are thus likely to help mediate the pathogenesis of *A. baumannii* [23]. Several extracellular proteins of *A. baumannii*, grown *in vitro*, have been identified by the use of different proteomic approaches [24, 25]. Such approaches have been used for membrane and cytoplasmic proteomic analysis of *A. baumannii* grown *in vitro* [26–30]. Comparative proteomic analysis has been performed with cells at three different stages of *in vitro* growth: exponential, late stationary phase and as biofilms [31]. Comparative proteomic analysis of a multidrug resistant strain with a drug-sensitive strain has also been performed [32]. *In vitro* culture conditions may stress bacteria (*e.g.* due to exhaustion of static nutrients, build-up of toxic bacterial by-products and limited physical space). However, simulated *in vitro* environments do not accurately reflect the protein profile within the lung, because *A. baumannii* must adjust to the environment inside the host lung (*e.g.*, CO_2_ concentrations, temperature and immune system) to be able to cause pneumonia.

The goal of this study was to improve our knowledge of the pathogenesis and virulence of *A. baumannii* by considering how the bacterium adapts to its host. In order to obtain an overview of host-pathogen-interactions during *A. baumannii* infections, we used, for the first time, a proteomics approach to compare the *ex vivo* proteomes of *A. baumannii* grown under different that mimic the physiological conditions that bacteria must face during *in vivo* host infection.

## Results

The objective of this study was to improve our knowledge of the pathogenesis and virulence of *A. baumannii* and to investigate how this *bacterium* adapts to its host. For this purpose, *A. baumannii* cells were cultured in LB (Luria-Bertani) medium with or without BALF (BALF model) and in Dulbecco’s Modified Eagle’s Medium (DMEM) medium with or without RAW 264.7 cells (macrophage model). The proteome composition of the *A. baumannii* cells in both models was analyzed in parallel, by using iTRAQ labelling and LC-MALDI-TOF/TOF to investigate the response of *A. baumannii* to exposure to macrophages/BALF (according to the scheme shown in Additional file 1).

### *Acinetobacter baumannii* caused consolidated pneumonia

BALF contains lipids, nucleic acids, secretomes of macrophages, lymphocytes, neutrophils, eosinophils, epithelial cells, pneumocytes, monocytes and basophils. Moreover, infection is capable of causing changes in concentrations, ratios and numbers of proteins detected in BALF. Therefore, to simulate conditions in the respiratory tract in response to *A. baumannii* infection, rats were inoculated with the bacterium and BALFs were obtained 21 h later. For histological assessment of *A. baumannii* infection of the airway, lung specimens were stained with haematoxylin and eosin and examined microscopically. Abscess formation, with extensive infiltration of polymorphonuclear leukocytes was noted [see Additional file 2].

### Quantitative (iTRAQ) data analysis

In the BALF model, *A. baumannii* was incubated simultaneously in LB and in LB supplemented with BALF (containing host soluble components present inside the lung 21 h after inoculation with *A. baumannii*) for 21 h at 37 °C and 5 % CO_2_ without shaking, in order to study the *A. baumannii* responses to soluble BALF components at the protein level by iTRAQ labelling and LC-MALDI/TOF analysis. Changes in the proteome of *A. baumannii* in response to soluble BALF components were examined. The rate of false positive results was estimated to be less than 1 % (with a confidence interval of 95 %) as the search was conducted in parallel with a decoy database using the “PSPEP on” mode, suggesting a high degree of confidence in the reported protein identifications. To evaluate the reproducibility and effectiveness of the iTRAQ experiments, we compared the proteins identified in each iTRAQ set. Overall, 8,310 unique peptides and 896 distinct proteins were detected with more than 95 % confidence in both iTRAQ sets. In biological replicate one, 730 proteins were identified and in biological replicate two, 853 proteins were identified. Only about 15.7 % of the proteins were identified with a single peptide. In total, 687 proteins (76.7 %) were identified in both biological replicates, and only 209 proteins (23.3 %) were unique for a single iTRAQ experiment. These results indicate the reliability of the iTRAQ identification of 687 proteins in all data sets. Compared with the control group, 111 identified proteins displayed significant changes in expression. In total, 50 proteins tended to be over-expressed (fold change >1.5, p <0.05) and 61 proteins tended to be under-expressed (fold change <0.5, p <0.05) relative to the control group (Table 1).

**Table 1:** Differentially expressed proteins under ex vivo conditions in BALF model

Protein description	AbH12O-A2 locus	Sec.^a^	BALF model/control^b^	BALF model/control *p*-value^c^
Pathogenesis and virulence				
Molecular chaperone DnaK	AIS05048.1	NO	1.77 (0.65)	0.027
Clp protease ClpX	AIS05265.1	NO	1.51 (0.61)	0.024
Amino acid ABC transporter substrate-binding protein (NfuA-like)	AIS05727.1	YES	2.38 (1.76)	0.031
Enoyl-CoA hydratase (PaaZ)	AIS06043.1	NO	0.30 (0.11)	0.012
Peptidase M16	AIS06751.1	NO	0.29 (0.16)	0.007
Aminopeptidase N	AIS06953.1	NO	0.39 (0.21)	0.023
Protein CsuC	AIS07073.1	YES	2.19 (0.76)	0.003
Protein CsuA	AIS07075.1	YES	1.87 (0.95)	0.036
Membrane protein (OmpA)	AIS07737.1	YES	1.51 (0.65)	0.015
Oligopeptidase A	AIS07885.1	NO	0.48 (0.11)	0.001
ABC transporter ATP-binding protein (YjjK)	AIS08062.1	NO	1.74 (0.61)	0.038
Amino acid metabolism and transport				
Aspartate-semialdehyde dehydrogenase	AIS05215.1	NO	0.25 (0.19)	0.038
2-Isopropylmalate synthase	AIS05260.1	NO	0.31 (0.11)	0.034
Ketol-acid reductoisomerase	AIS05310.1	NO	0.31 (0.05)	0.027
Ornithine carbamoyltransferase	AIS06867.1	NO	0.40 (0.17)	0.044
Aspartate aminotransferase	AIS07065.1	NO	0.08 (0.07)	0.000
Glutamate synthase	AIS08029.1	NO	0.39 (0.20)	0.025
Carbohydrate metabolism and transport				
Phosphoglyceromutase	AIS05033.1	NO	0.26 (0.14)	0.020
Phosphoenolpyruvate synthase	AIS07024.1	NO	0.19 (0.04)	0.000
Glucose dehydrogenase	AIS07744.1	NO	0.19 (0.13)	0.009
Cell cycle control and mitosis				
Cell division inhibitor MinD	AIS05622.1	NO	1.63 (1.55)	0.000
tRNA uridine 5-carboxymethylaminomethyl modification protein	AIS07041.1	NO	2.70 (1.89)	0.025
Cell division protein FtsZ	AIS08195.1	NO	4.61 (1.68)	0.001
Cell wall/membrane/envelope biogenesis				
Membrane protein(OmpW)	AIS05087.1	YES	2.25 (0.89)	0.032
UDP-N-acetylglucosamine 1-carboxyvinyltransferase (MurA)	AIS05387.1	NO	2.27 (1.47)	0.002
UDP-N-acetylmuramate-alanine ligase (MurC)	AIS08199.1	NO	99.08 (0.00)	0.020
Coenzyme metabolism				
Coproporphyrinogen III oxidase	AIS07950.1	NO	0.31 (0.13)	0.004
3-Phosphoglycerate dehydrogenase	AIS07996.1	NO	0.38 (0.09)	0.016
Defense mechanisms (cellular processes and signaling)				
RND transporter	AIS04803.1	YES	0.30 (0.22)	0.004
Energy production and conversion				
Malate dehydrogenase	AIS04961.1	NO	0.45 (0.23)	0.038
Inorganic pyrophosphatase	AIS05013.1	YES	2.83 (1.43)	0.030
Aconitate hydratase	AIS05319.1	NO	0.21 (0.16)	0.000
NAD(P) transhydrogenase subunit alpha	AIS05328.1	NO	2.49 (1.37)	0.041
NADH dehydrogenase	AIS05456.1	NO	0.44 (0.17)	0.012
Cytochrome C oxidase subunit II	AIS07026.1	NO	0.21 (0.23)	0.016
Malic enzyme	AIS07199.1	NO	0.46 (0.10)	0.041
Isocitrate dehydrogenase	AIS07371.1	NO	0.29 (0.05)	0.004
Phosphoenolpyruvate carboxykinase	AIS07529.1	NO	2.23 (0.66)	0.000
Succinyl-CoA synthetase subunit beta	AIS07559.1	NO	0.41 (0.06)	0.035
Energy production and conversion				
2-Oxoglutarate dehydrogenase	AIS07562.1	NO	0.20 (0.04)	0.000
Succinate dehydrogenase	AIS07563.1	NO	1.98 (0.64)	0.017
Type II citrate synthase	AIS07567.1	NO	0.22 (0.05)	0.002
Oxidoreductase	AIS07724.1	NO	0.31 (0.22)	0.001
Malate dehydrogenase	AIS07865.1	NO	0.47 (0.06)	0.040
Acetyl-CoA hydrolase	AIS08069.1	NO	2.05 (0.67)	0.008
Pyruvate dehydrogenase	AIS08191.1	NO	0.36 (0.06)	0.000
Unknown function				
Hypothetical protein	AIS05481.1	YES	0.07 (0.10)	0.013
Peptidoglycan-binding protein LysM	AIS05521.1	YES	0.05 (0.03)	0.003
Hypothetical protein	AIS06125.1	YES	0.17 (0.07)	0.027
DNA-binding protein	AIS07086.1	YES	0.08 (0.07)	0.006
Peptidase	AIS07285.1	YES	4.57 (3.14)	0.000
Glyoxalase	AIS07740.1	YES	0.39 (0.45)	0.015
Hypothetical protein	AIS07742.1	YES	1.84 (1.06)	0.048
General functional prediction only (typically, prediction of biochemical activity)				
Alpha/beta hydrolase	AIS06658.1	YES	0.09 (0.07)	0.001
GTPase obg	AIS07390.1	NO	2.58 (1.05)	0.025
Hypothetical protein	AIS07569.1	NO	3.47 (1.83)	0.027
Inorganic ion transport and metabolism				
Bacterioferritin	AIS05502.1	NO	0.25 (0.18)	0.013
Hydroperoxidase	AIS06129.1	YES	0.26 (0.10)	0.000
Inorganic ion transport and metabolism				
Superoxide dismutase	AIS07203.1	YES	0.49 (0.14)	0.013
Sulfurtransferase	AIS07821.1	NO	0.37 (0.28)	0.006
Intracellular trafficking and secretion				
RNA-binding protein	AIS05494.1	NO	2.07 (0.97)	0.001
Lipid metabolism				
3-Ketoacyl-CoA thiolase	AIS05097.1	NO	0.35 (0.18)	0.049
Multifunctional fatty acid oxidation complex subunit alpha	AIS05098.1	NO	0.29 (0.08)	0.000
Acetyl-CoA carboxylase	AIS05368.1	NO	2.01 (0.67)	0.000
3-Hydroxyacyl-CoA dehydrogenase	AIS06051.1	NO	0.32 (0.22)	0.023
Beta-ketoadipyl CoA thiolase	AIS06052.1	NO	0.28 (0.10)	0.000
3-Methylcrotonyl-CoA carboxylase	AIS06111.1	NO	0.11 (0.04)	0.002
Short-chain dehydrogenase	AIS06130.1	YES	0.04 (0.05)	0.040
Acetyl-CoA acetyltransferase	AIS06469.1	YES	0.09 (0.13)	0.027
Acetyl-CoA carboxylase	AIS06900.1	NO	0.42 (0.17)	0.001
3-Hydroxy-2-methylbutyryl-CoA dehydrogenase	AIS07009.1	NO	0.25 (0.18)	0.031
Acetyl-CoA acetyltransferase	AIS07739.1	YES	0.24 (0.26)	0.017
Acyl-CoA dehydrogenase	AIS07780.1	NO	0.22 (0.16)	0.000
Nucleotide metabolism and transport				
GMP synthase	AIS04952.1	NO	0.31 (0.17)	0.000
Formyltetrahydrofolate deformylase	AIS05238.1	NO	1.64 (1.02)	0.012
Deoxyuridine 5′-triphosphate nucleotidohydrolase	AIS05628.1	NO	4.45 (6.37)	0.039
Dihydroorotase	AIS05812.1	NO	1.79 (1.15)	0.043
Nucleotide metabolism and transport				
Orotate phosphoribosyltransferase	AIS08207.1	NO	2.73 (1.78)	0.004
Post-translational modification, protein turnover, chaperone functions				
Peroxidase	AIS07758.1	YES	0.25 (0.21)	0.033
Replication and repair				
Chromosomal replication initiation protein	AIS04811.1	NO	81.66 (31.26)	0.017
DNA gyrase subunit A	AIS07481.1	NO	0.09 (0.16)	0.004
Transcription				
Antitermination protein NusG	AIS05075.1	NO	2.00 (0.81)	0.010
DNA-directed RNA polymerase subunit beta	AIS05080.1	NO	0.26 (0.05)	0.000
DNA-directed RNA polymerase subunit beta’	AIS05081.1	NO	0.10 (0.04)	0.000
RNA polymerase sigma factor RpoD	AIS07572.1	NO	1.92 (1.40)	0.042
Translation				
Isoleucine-tRNA ligase	AIS04850.1	NO	0.28 (0.09)	0.001
Arginine-tRNA ligase	AIS04960.1	NO	0.47 (0.16)	0.019
50S ribosomal protein L1	AIS05077.1	YES	3.40 (0.93)	0.000
30S ribosomal protein S15	AIS05149.1	NO	15.56 (6.87)	0.045
50S ribosomal protein L28	AIS05235.1	YES	1.85 (0.68)	0.042
Leucyl-tRNA synthetase	AIS05306.1	NO	0.39 (0.16)	0.029
Threonyl-tRNA synthetase	AIS05352.1	NO	0.09 (0.16)	0.000
30S ribosomal protein S7	AIS05609.1	NO	9.91 (4.58)	0.000
Elongation factor Tu	AIS05611.1	NO	0.34 (0.05)	0.002
Cysteinyl-tRNA synthetase	AIS05938.1	NO	0.18 (0.11)	0.015
30S ribosomal protein S6	AIS07031.1	NO	3.56 (0.80)	0.004
Translation				
50S ribosomal protein L9	AIS07033.1	NO	4.70 (2.06)	0.000
30S ribosomal protein S21	AIS07108.1	YES	4.33 (3.26)	0.040
50S ribosomal protein L21	AIS07586.1	NO	4.45 (2.38)	0.029
Valyl-tRNA synthetase	AIS07599.1	NO	0.17 (0.14)	0.000
30S ribosomal protein S9	AIS07845.1	YES	6.19 (2.19)	0.005
50S ribosomal protein L17	AIS07895.1	YES	15.14 (7.44)	0.025
30S ribosomal protein S11	AIS07898.1	YES	3.40 (1.58)	0.002
50S ribosomal protein L15	AIS07902.1	NO	5.06 (1.54)	0.000
50S ribosomal protein L6	AIS07906.1	YES	5.35 (2.12)	0.000
30S ribosomal protein S8	AIS07907.1	YES	7.52 (1.56)	0.007
50S ribosomal protein L5	AIS07909.1	NO	7.18 (2.38)	0.000
50S ribosomal protein L14	AIS07911.1	NO	5.20 (1.87)	0.002
50S ribosomal protein L22	AIS07916.1	NO	2.03 (0.76)	0.012
50S ribosomal protein L23	AIS07919.1	YES	1.77 (0.58)	0.043
Aminoglycoside phosphotransferase	AIS08113.1	NO	2.88 (4.81)	0.015

The protein profiles produced by *A. baumannii* grown in modified BALF were performed using iTRAQ reagents and LC-MS/MS. Differential expression was defined by a relative abundance ratio >1.5 and <0.5

^a^Secretion prediction are based on (SignalP 4.1 (http://www.cbs.dtu.dk/services/SignalP/), Phobius (http://phobius.sbc.su.se/), PrediSi (http://www.predisi.de/), TatP 1.0 (www.cbs.dtu.dk/services/TatP-1.0), Tatfind 1.4 (http://signalfind.org/tatfind.html), SecretomeP 2.0 (www.cbs.dtu.dk/services/SecretomeP), TMHMM (http://www.cbs.dtu.dk/services/TMHMM/), DAS-TMfilter (http://www.enzim.hu/DAS/DAS.html), LipoP 1.0 (www.cbs.dtu.dk/services/LipoP), DOLOP (http://www.mrc-lmb.cam.ac.uk/genomes/dolop/), and LIPO (http://services.cbu.uib.no/tools/lipo))

^b^Average relative protein expression level ratio in sample and control, with the standard deviation in parentheses, quantified by Protein Pilot 4.0 software (ABSciex).

^c^Determined by Student’s *t* test. Values of less than 0.05 are considered significant

In the macrophage model, infection of macrophages was induced with *A. baumannii* (multiplicity of infection 3). Control and infected cells were incubated simultaneously for 21 h at 37 °C and 5 % CO_2,_ without shaking, in order to study the *A. baumannii* responses to immune system cells at the protein level by iTRAQ labelling and LC-MALDI/TOF analysis. Changes in the proteome of *A. baumannii* in response to RAW 264.7 cells were also examined. In comparison with the control group, 97 identified proteins displayed significant changes in expression. A total of 76 proteins displayed increased expression levels (fold change >1.5, p <0.05) and 21 proteins displayed decreased expression levels (fold change <0.5, p <0.05) relative to the control group (Table 2).

**Table 2:** Differentially expressed proteins under ex vivo conditions in macrophage model

Protein description	AbH12O-A2 locus	Sec.^a^	macrophage model/ control^b^	macrophage model/ control *p*-value^c^
Pathogenesis and virulence				
Nucleotidyl transferase	AIS04894.1	NO	2.83 (0.60)	0.013
Trigger factor	AIS05263.1	NO	3.37 (0.37)	0.016
ATPase AAA (PaaA)	AIS06044.1	NO	5.50 (4.44)	0.028
Siderophore achromobactin biosynthesis proteína AcsC	AIS06377.1	NO	0.44 (0.17)	0.000
Cyclophilin (PPIase)	AIS06968.1	NO	2.83 (0.87)	0.041
Protein CsuC	AIS07073.1	YES	0.16 (0.06)	0.004
Protein CsuA	AIS07075.1	YES	0.31 (0.22)	0.040
Metallopeptidase	AIS07555.1	YES	0.20 (0.16)	0.028
Membrane protein (OmpA)	AIS07737.1	YES	7.80 (2.78)	0.049
Oligopeptidase A	AIS07885.1	NO	4.83 (0.88)	0.006
ABC transporter ATP-binding protein (YjjK)	AIS08062.1	NO	2.33 (0.77)	0.046
Amino acid metabolism and transport				
Aspartate aminotransferase	AIS07065.1	NO	0.39 (0.18)	0.010
Serine hydroxymethyltransferase	AIS07167.1	NO	2.54 (0.44)	0.043
Carbamoyl-phosphate synthase	AIS07544.1	NO	2.75 (1.74)	0.035
4-Hydroxyphenylpyruvate dioxygenase	AIS08279.1	NO	4.74 (1.15)	0.000
Carbohydrate metabolism and transport				
Phosphoglyceromutase	AIS05033.1	NO	3.94 (1.06)	0.032
Glyceraldehyde-3-phosphate dehydrogenase	AIS07393.1	NO	3.08 (0.66)	0.016
Cell cycle control and mitosis				
Cell division protein FtsA	AIS08196.1	NO	4.74 (7.32)	0.002
Cell wall/membrane/envelope biogenesis				
Membrane protein	AIS06856.1	YES	8.02 (7.90)	0.006
Racemase	AIS07059.1	NO	0.20 (0.13)	0.037
UDP-N-acetylmuramate-alanine ligase (MurC)	AIS08199.1	NO	8.17 (3.95)	0.040
Coenzyme metabolism				
Pantoate-beta-alanine ligase (PanC)	AIS05347.1	NO	6.08 (8.59)	0.010
3-Phosphoglycerate dehydrogenase	AIS07996.1	NO	3.77 (0.88)	0.014
Defense mechanisms (cellular processes and signaling)				
Beta-lactamase	AIS07280.1	YES	2.09 (0.41)	0.049
ABC transporter	AIS07290.1	NO	0.39 (0.18)	0.037
Energy production and conversion				
ATP synthase subunit B	AIS04971.1	NO	5.50 (2.54)	0.044
ATP synthase F0F1 subunit beta	AIS04975.1	NO	3.63 (0.48)	0.002
Bifunctional aconitate hydratase 2/2-methylisocitrate dehydratase	AIS06989.1	NO	3.16 (0.31)	0.002
Isocitrate dehydrogenase	AIS07371.1	NO	4.57 (0.55)	0.002
Electron transfer flavoprotein subunit beta	AIS07482.1	YES	5.65 (0.89)	0.005
Dihydrolipoamide succinyltransferase	AIS07561.1	NO	2.44 (0.45)	0.036
Fumarate reductase	AIS07564.1	NO	3.08 (0.59)	0.010
Acetyl-CoA hydrolase	AIS08069.1	NO	4.74 (1.68)	0.040
NADPH:quinone oxidoreductase	AIS08156.1	NO	15.42 (16.20)	0.034
Dihydrolipoamide acetyltransferase	AIS08190.1	YES	3.08 (0.73)	0.010
Unknown function				
Hypothetical protein	AIS05430.1	NO	19.23 (19.19)	0.001
Hypothetical protein	AIS05481.1	YES	0.17 (0.08)	0.000
Unknown function				
Hypothetical protein	AIS05936.1	YES	0.23 (0.13)	0.006
DNA-binding protein	AIS07086.1	YES	0.24 (0.17)	0.037
Hypothetical protein	AIS07091.1	NO	0.21 (0.12)	0.000
DcaP-like protein	AIS07608.1	YES	11.07 (6.85)	0.023
Glyoxalase	AIS07740.1	YES	0.30 (0.23)	0.036
Hypothetical protein	AIS07873.1	YES	0.42 (0.16)	0.011
Inorganic ion transport and metabolism				
Hydroperoxidase	AIS06129.1	YES	0.35 (0.13)	0.008
ABC transporter permease	AIS07421.1	YES	8.17 (3.60)	0.034
Lipid metabolism				
3-Oxoacyl-ACP reductase	AIS05519.1	YES	2.68 (0.94)	0.038
Enoyl-CoA hydratase	AIS06112.1	NO	0.17 (0.24)	0.004
Short-chain dehydrogenase	AIS06130.1	YES	0.10 (0.07)	0.032
Nucleotide metabolism and transport				
N5-carboxyaminoimidazole ribonucleotide mutase	AIS04832.1	NO	4.02 (2.98)	0.010
Ribose-phosphate pyrophosphokinase	AIS05530.1	NO	4.61 (2.76)	0.000
Deoxyuridine 5′-triphosphate nucleotidohydrolase	AIS05628.1	NO	0.28 (0.32)	0.032
Orotidine 5′-phosphate decarboxylase	AIS06325.1	NO	0.19 (0.21)	0.032
Adenylosuccinate lyase	AIS07333.1	NO	6.31 (2.68)	0.047
Xanthine phosphoribosyltransferase	AIS07874.1	NO	2.42 (0.69)	0.048
Inosine-5-monophosphate dehydrogenase	AIS08184.1	NO	3.94 (0.81)	0.006
Post-translational modification, protein turnover, chaperone functions				
Molecular chaperone DnaK	AIS04838.1	NO	2.13 (0.15)	0.000
Post-translational modification, protein turnover, chaperone functions				
Osmotically inducible protein C	AIS04957.1	NO	3.16 (1.14)	0.029
Replication and repair				
DNA repair protein	AIS04967.1	YES	3.87 (1.19)	0.033
DNA polymerase I	AIS05372.1	NO	0.31 (0.34)	0.038
Secondary metabolites: biosynthesis, transport and catabolism				
mRNA 3′-end processing factor	AIS06680.1	NO	0.10 (0.19)	0.027
Transcription				
DNA-directed RNA polymerase subunit beta’	AIS05081.1	NO	2.86 (0.42)	0.025
Transcription elongation factor NusA	AIS05126.1	NO	3.53 (0.94)	0.036
Transcription termination factor Rho	AIS05365.1	NO	3.60 (1.54)	0.025
DNA-binding protein	AIS07045.1	NO	9.73 (6.82)	0.029
DNA-directed RNA polymerase subunit alpha	AIS07896.1	NO	3.28 (0.46)	0.001
Translation				
Tyrosyl-tRNA synthetase	AIS04798.1	NO	3.37 (0.97)	0.009
23S rRNA methyltransferase	AIS05117.1	NO	3.37 (4.80)	0.037
Alanyl-tRNA synthetase	AIS05862.1	NO	3.05 (1.16)	0.004
30S ribosomal protein S20	AIS06347.1	YES	3.77 (2.31)	0.026
Ribosome-recycling factor	AIS06861.1	NO	3.91 (1.42)	0.045
Peptide chain release factor 1	AIS07012.1	NO	0.34 (0.27)	0.021
30S ribosomal protein S6	AIS07031.1	NO	6.92 (1.66)	0.000
30S ribosomal protein S18	AIS07032.1	NO	22.70 (16.56)	0.028
50S ribosomal protein L9	AIS07033.1	NO	8.17 (3.02)	0.002
30S ribosomal protein S2	AIS07184.1	NO	3.87 (1.07)	0.040
Translation				
Tryptophanyl-tRNA synthetase	AIS07557.1	NO	8.09 (4.24)	0.023
50S ribosomal protein L27	AIS07585.1	YES	10.28 (8.73)	0.027
Aspartyl-tRNA synthetase	AIS07787.1	NO	5.92 (1.79)	0.038
30S ribosomal protein S9	AIS07845.1	YES	5.86 (1.38)	0.009
50S ribosomal protein L17	AIS07895.1	YES	9.64 (4.81)	0.003
30S ribosomal protein S4	AIS07897.1	NO	13.30 (3.19)	0.010
50S ribosomal protein L15	AIS07902.1	NO	5.92 (1.17)	0.000
30S ribosomal protein S5	AIS07904.1	YES	4.61 (5.00)	0.003
50S ribosomal protein L18	AIS07905.1	YES	9.04 (10.58)	0.033
50S ribosomal protein L6	AIS07906.1	YES	7.59 (1.58)	0.037
30S ribosomal protein S8	AIS07907.1	YES	10.76 (2.98)	0.011
50S ribosomal protein L5	AIS07909.1	NO	7.73 (2.20)	0.000
50S ribosomal protein L24	AIS07910.1	YES	4.25 (0.70)	0.038
50S ribosomal protein L14	AIS07911.1	NO	11.07 (12.57)	0.002
30S ribosomal protein S17	AIS07912.1	NO	12.36 (3.91)	0.048
50S ribosomal protein L16	AIS07914.1	YES	29.11 (22.95)	0.038
30S ribosomal protein S3	AIS07915.1	NO	6.73 (3.82)	0.026
50S ribosomal protein L22	AIS07916.1	NO	1.89 (0.85)	0.035
50S ribosomal protein L2	AIS07918.1	YES	7.87 (3.98)	0.038
50S ribosomal protein L23	AIS07919.1	YES	4.45 (1.04)	0.018
50S ribosomal protein L4	AIS07920.1	NO	14.45 (7.02)	0.002
Translation				
50S ribosomal protein L3	AIS07921.1	NO	6.25 (1.29)	0.042

The protein profiles produced by *A. baumannii* grown in the presence of macrophages were performed using iTRAQ reagents and LC-MS/MS. Differential expression was defined by a relative abundance ratio >1.5 and <0.5

^a^Secretion prediction are based on (SignalP 4.1 (http://www.cbs.dtu.dk/services/SignalP/), Phobius (http://phobius.sbc.su.se/), PrediSi (http://www.predisi.de/), TatP 1.0 (www.cbs.dtu.dk/services/TatP-1.0), Tatfind 1.4 (http://signalfind.org/tatfind.html), SecretomeP 2.0 (www.cbs.dtu.dk/services/SecretomeP), TMHMM (http://www.cbs.dtu.dk/services/TMHMM/), DAS-TMfilter (http://www.enzim.hu/DAS/DAS.html), LipoP 1.0 (www.cbs.dtu.dk/services/LipoP), DOLOP (http://www.mrc-lmb.cam.ac.uk/genomes/dolop/), and LIPO (http://services.cbu.uib.no/tools/lipo))

^b^Average relative protein expression level ratio in sample and control, with the standard deviation in parentheses, quantified by Protein Pilot 4.0 software (ABSciex).

^c^Determined by Student’s *t* test. Values of less than 0.05 are considered significant

**Fig. 1:**
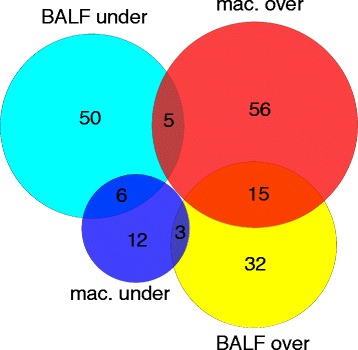
Venn diagram showing the number and relationship between *A. baumannii* proteins that were differentially expressed in comparison of 2 ex vivo models. Circles represent the set of over-expressed proteins (red) and under-expressed proteins (dark blue) in the macrophage model and the set of over-expressed proteins (yellow) and under-expressed proteins (light blue) in the BALF model. The number of proteins differentially expressed is indicated in each set or subset

Overall, 179 differentially expressed proteins were identified in both models. 111 proteins were modulated in the BALF model and 97 proteins were modulated in the macrophage model. In the BALF model, 45.0 % of the differentially expressed proteins were over-expressed. In the macrophage model, 78.4 % of the modulated proteins were over-expressed. Of the 179 modulated proteins, 16.2 % (29 proteins) were differentially expressed in both models, 51.7 % (15 proteins) were overexpressed, 20.7 % (6 proteins) were underexpressed and 27.6 % (8 proteins) were opposite (over-expressed in a model and under-expressed in the other model) (Fig. 1, Table 3).

**Table 3:** Differentially expressed proteins in both ex vivo models

Protein description	AbH12O-A2 locus	Sec.^a^	BALF model/control^b^	BALF model/control *p*-value^c^	macrophage model/control^b^	macrophage model/control *p*-value^c^
Pathogenesis and virulence						
Protein CsuC	AIS07073.1	YES	2.19 (0.76)	0.003	0.16 (0.06)	0.004
Protein CsuA	AIS07075.1	YES	1.87 (0.95)	0.036	0.31 (0.22)	0.040
Membrane protein (OmpA)	AIS07737.1	YES	1.51 (0.65)	0.015	7.80 (2.78)	0.049
Oligopeptidase A	AIS07885.1	NO	0.48 (0.11)	0.001	4.83 (0.88)	0.006
ABC transporter ATP-binding protein (YjjK)	AIS08062.1	NO	1.74 (0.61)	0.038	2.33 (0.77)	0.046
Amino acid metabolism and transport						
Aspartate aminotransferase	AIS07065.1	NO	0.08 (0.07)	0.000	0.39 (0.18)	0.010
Carbohydrate metabolism and transport						
Phosphoglyceromutase	AIS05033.1	NO	0.26 (0.14)	0.020	3.94 (1.06)	0.032
Cell wall/membrane/envelope biogenesis						
UDP-N-acetylmuramate-alanine ligase (MurC)	AIS08199.1	NO	99.08 (0.00)	0.020	8.17 (3.95)	0.040
Coenzyme metabolism						
3-Phosphoglycerate dehydrogenase	AIS07996.1	NO	0.38 (0.09)	0.016	3.77 (0.88)	0.014
Energy production and conversion						
Isocitrate dehydrogenase	AIS07371.1	NO	0.29 (0.05)	0.004	4.57 (0.55)	0.002
Acetyl-CoA hydrolase	AIS08069.1	NO	2.05 (0.67)	0.008	4.74 (1.68)	0.040
Unknown function						
Hypothetical protein	AIS05481.1	YES	0.07 (0.10)	0.013	0.17 (0.08)	0.000
DNA-binding protein	AIS07086.1	YES	0.08 (0.07)	0.006	0.24 (0.17)	0.037
Glyoxalase	AIS07740.1	YES	0.39 (0.45)	0.015	0.30 (0.23)	0.036
Inorganic ion transport and metabolism						
Hydroperoxidase	AIS06129.1	YES	0.26 (0.10)	0.000	0.35 (0.13)	0.008
Lipid metabolism						
Short-chain dehydrogenase	AIS06130.1	YES	0.04 (0.05)	0.040	0.10 (0.07)	0.032
Nucleotide metabolism and transport						
Deoxyuridine 5′-triphosphate nucleotidohydrolase	AIS05628.1	NO	4.45 (6.37)	0.039	0.28 (0.32)	0.032
Transcription						
DNA-directed RNA polymerase subunit beta’	AIS05081.1	NO	0.10 (0.04)	0.000	2.86 (0.42)	0.025
Translation						
30S ribosomal protein S6	AIS07031.1	NO	3.56 (0.80)	0.004	6.92 (1.66)	0.000
50S ribosomal protein L9	AIS07033.1	NO	4.70 (2.06)	0.000	8.17 (3.02)	0.002
30S ribosomal protein S9	AIS07845.1	YES	6.19 (2.19)	0.005	5.86 (1.38)	0.009
50S ribosomal protein L17	AIS07895.1	YES	15.14 (7.44)	0.025	9.64 (4.81)	0.003
50S ribosomal protein L15	AIS07902.1	NO	5.06 (1.54)	0.000	5.92 (1.17)	0.000
50S ribosomal protein L6	AIS07906.1	YES	5.35 (2.12)	0.000	7.59 (1.58)	0.037
30S ribosomal protein S8	AIS07907.1	YES	7.52 (1.56)	0.007	10.76 (2.98)	0.011
50S ribosomal protein L5	AIS07909.1	NO	7.18 (2.38)	0.000	7.73 (2.20)	0.000
50S ribosomal protein L14	AIS07911.1	NO	5.20 (1.87)	0.002	11.07 (12.57)	0.002
50S ribosomal protein L22	AIS07916.1	NO	2.03 (0.76)	0.012	1.89 (0.85)	0.035
50S ribosomal protein L23	AIS07919.1	YES	1.77 (0.58)	0.043	4.45 (1.04)	0.018

The protein profiles produced by *A. baumannii* grown in modified BALF and in the presence of macrophages were performed using iTRAQ reagents and LC-MS/MS. Differential expression was defined by a relative abundance ratio >1.5 and <0.5

^a^Secretion prediction are based on (SignalP 4.1 (http://www.cbs.dtu.dk/services/SignalP/), Phobius (http://phobius.sbc.su.se/), PrediSi (http://www.predisi.de/), TatP 1.0 (www.cbs.dtu.dk/services/TatP-1.0), Tatfind 1.4 (http://signalfind.org/tatfind.html), SecretomeP 2.0 (www.cbs.dtu.dk/services/SecretomeP), TMHMM (http://www.cbs.dtu.dk/services/TMHMM/), DAS-TMfilter (http://www.enzim.hu/DAS/DAS.html), LipoP 1.0 (www.cbs.dtu.dk/services/LipoP), DOLOP (http://www.mrc-lmb.cam.ac.uk/genomes/dolop/), and LIPO (http://services.cbu.uib.no/tools/lipo))

^b^Average relative protein expression level ratio in sample and control, with the standard deviation in parentheses, quantified by Protein Pilot 4.0 software (ABSciex).

^c^Determined by Student’s *t* test. Values of less than 0.05 are considered significant

### Analysis of modulated proteins

**Fig. 2:**
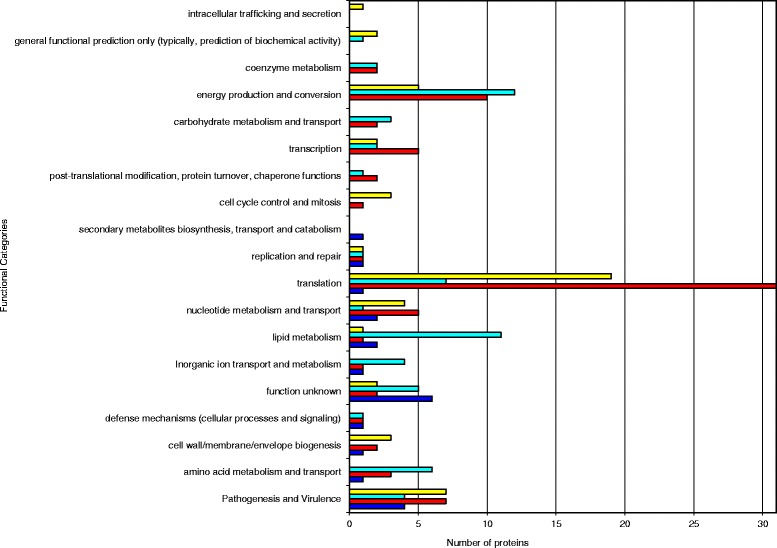
Distribution of differentially expressed proteins in *A. baumannii* following ex vivo incubation according to functional categories. The stacked bar chart shows the number of over-expressed proteins (red) and under-expressed proteins (dark blue) in the macrophage model and the number of over-expressed proteins (yellow) and under-expressed proteins (light blue) in the BALF model in each functional category

According to their predicted biological functions, the differentially expressed proteins were divided into 19 groups (Fig. 2). The largest groups consisted of proteins involved in translation (58), followed by the proteins involved in energy production and conversion (27) and by proteins involved in pathogenesis and virulence (23). Most of the proteins over-expressed in the BALF model were involved in translation (38.0 %), followed by pathogenesis and virulence (14.0 %), and energy production and conversion (10.0 %). In the macrophage model, most were involved in translation (40.8 %), followed by energy production and conversion (13.2 %), and pathogenesis and virulence (10.5 %). Most of the proteins that were under-expressed in the BALF model were involved in energy production and conversion (19.7 %) and in the macrophage model most were proteins of unknown function (28.6 %).

Of the 29 proteins differentially expressed in both models, most were involved in translation (37.9 %), followed by pathogenesis and virulence (17.2 %) and by unknown function (10.3 %). Most of the over-expressed proteins were involved in translation, whereas most of the under-expressed proteins were proteins of unknown function, and 50.0 % of opposite proteins were associated with metabolism (Table 3).

### RT-PCR

We selected some proteins for validation of the proteomic data by RT-PCR analysis. The candidate proteins, *NfuA*-like, *YjjK*, *CsuC*, *OmpW*, *OmpA*, *ClpX*, *DNA repair*, *PaaA* and *PpiA* were selected on the basis of their differential expression as representative of pathogenesis and virulence, cell wall/membrane/envelope biogenesis and other functional categories.

#### BALF model

**Fig. 3:**
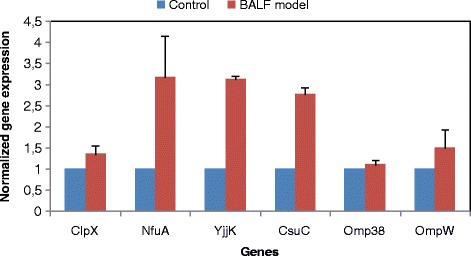
RT-PCR analysis of different genes coding for differentially expressed proteins in controls and samples (BALF model). All expression results were normalized relative to *RpoB* by the 2^−ΔΔCt^ method. For all genes, relative mRNA expression is presented as a fold-change value relative to the control

RT-PCR analysis was applied to 6 genes coding for differentially expressed proteins to confirm the findings of the proteomic analysis. Expression of most, but not all, of these genes paralleled expression of the corresponding proteins revealed by proteomic analysis. Four (*NfuA*-like, *YjjK*, *CsuC* and *OmpW*) of the six selected genes were over-expressed at the transcriptional level in the sample relative to the control which is consistent with the results of protein expression. However, there was a slight inconsistency between the translational and transcriptional levels of *OmpA* and *ClpX*. Expression of *OmpA* and *ClpX* mRNA revealed similar patterns in the sample relative to the control, despite the increased levels of the respective mRNAs (Fig. 3, Table 1).

#### Macrophage model

**Fig. 4:**
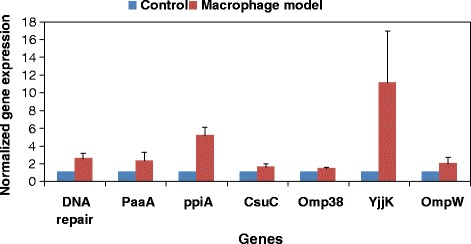
RT-PCR analysis of different genes coding for differentially expressed proteins in controls and samples (macrophage model). All expression results were normalized relative to *RpoB* by the 2^−ΔΔCt^ method. For all genes, relative mRNA expression is presented as a fold-change value relative to the control

RT-PCR analysis of 7 genes coding for differentially expressed proteins was performed to confirm the results of the proteomic analysis. Expression of most, but not all, of the genes paralleled the expression of the corresponding proteins revealed by proteomic analysis. Five (*DNA repair*, *PaaA*, *PpiA*,*YjjK* and *OmpW*) of the seven selected genes were over-expressed at the transcriptional level in the sample, relative to the control, which is consistent with the results of protein expression. However, there was a slight inconsistency between the translational and transcriptional levels of *OmpA* and an inconsistency between the translational and transcriptional levels of *CsuC*. The mRNA expression of *OmpA* revealed similar patterns in the sample, relative to the control, despite the increased levels of the respective mRNAs. The mRNA expression of *CsuC* was 1.6 times higher and the protein abundance was more than six times lower in the sample than in the control (Fig. 4, Table 2), probably because of post-transcriptional regulation [33].

Comparison of the results obtained by iTRAQ labelling-LC-MALDI/TOF and RT-PCR showed that the changes observed in protein abundance between samples of both *ex vivo* models and controls were also reflected in the mRNA levels.

## Discussion

The host activated response to *Acinetobacter baumannii* is not well understood. Some studies have described changes in *A. baumannii* gene expression under *in vivo*-mimicking conditions. However, most of these studies focused on transcriptional changes of one or a few genes of interest, mostly under iron limiting conditions [34–38]. A microarray study defined the expression properties of *A. baumannii* during growth in human serum and demonstrated significant over-expression of iron acquisition systems, of genes associated with epithelial cell adherence, DNA uptake and of numerous putative drug efflux pumps [39]. At the protein level, comparative analysis of total lysate and outer membrane fractions isolated from *A. baumannii* cultured under iron-rich and iron-chelated conditions led to identification of 58 modulated proteins [40]. There remains an alarmingly large gap in our knowledge of the pathogenesis and nature of *A. baumannii* virulence. A better understanding of the adaptation of *A. baumannii* to the host and its molecular pathogenesis is essential for the development of new therapeutic and diagnostic methods. Research studies that address the *A. baumannii* proteome *in vivo* and the proteome that is produced in the presence of fluid or host cells are lacking. In contrast to the numerous proteomic studies of *A. baumannii* cultivated *in vitro* laboratory settings, only one microarray study has been carried out *ex vivo*, and no *ex vivo* or *in vivo* proteomic studies have been performed. This is because of technical limitations, which severely limit the number of recovered bacteria, and the potential contamination of sample preparations by host cell nucleic acids or proteins.

To identify putative pathogenesis and virulence factors that mediate *A. baumannii* pathogenesis, we used 2 *ex vivo* models and a quantitative proteomics approach. We provide evidence that expression of 179 *A. baumannii* proteins is affected by the host. Comparison of both models showed that 83 % of the proteins detected were grouped in the same category in both models (under-expressed, over-expressed and un-modulated) and that most of the proteins were unmodulated, which indicates that the host proteins may target and regulate a specific small group of *A. baumannii* proteins. The modulated proteins possess diverse functions (Fig. 2, Table 1 and Table 2), which suggests a complex interaction between the bacterium and the host. Thus, in the BALF model, 50 of the proteins identified were over-expressed, whereas 61 were under-expressed. Some of these functional groups are clearly over-expressed for functioning in cell wall/membrane/envelope biogenesis, nucleotide metabolism and transport and cell cycle control and mitosis. Other important biological processes, such as pathogenesis and virulence and translation, were mainly over-expressed. However, other metabolic proteins involved in, *e.g.*, amino acid metabolism and lipid metabolism were under-expressed. In the macrophage model, 76 of the proteins identified were over-expressed, whereas 21 were under-expressed. Some of these functional groups are clearly over-expressed for functioning in translation, transcription and energy production and conversion. Other important biological processes, such as pathogenesis and virulence and nucleotide metabolism and transport, were mainly over-expressed. However, other functional groups involved in lipid metabolism were under-expressed. Although both models yielded generally similar results, some differences in the findings are not surprising, for the following reasons. First, different rodents species were used as hosts. Moreover, some of the major influencing factors identified in the BALF model include nutritional differences between samples (LB broth+lipids, nucleic acids, peptides and proteins) and controls (LB) and stress resulting from host inflammatory response (proteins important for immunity and host defence functions, such as surfactant protein A and surfactant protein D). However, in the macrophage model, these factors may compete with macrophages for available nutrients and stress resulting from interaction with a single host cell type.

Proteomic comparison of the levels of protein expression in the models revealed that 82 proteins were only modulated by BALF, 68 proteins were only modulated by macrophages and 29 proteins were modulated by both. Of these 29 proteins, 15 were over-expressed, 6 proteins were under-expressed and 8 proteins were opposite (Fig. 1). Interestingly, 50.0 % of opposite proteins were associated with metabolism (phosphoglyceromutase, 3-phosphoglycerate dehydrogenase, isocitrate dehydrogenase and deoxyuridine 5′-triphosphate nucleotidohydrolase). This is probably due to differences in the concentration of available nutrients in both models and to competition with macrophages for available nutrients in the macrophage model. Over-expressed amino acid catabolism was also observed in the macrophage model and under-expressed amino acid catabolism in the BALF model (Table 3), indicating that amino acids constitute a major source of nitrogen for *A. baumannii* in the macrophage model. The availability of amino acids is probably also partly due to uptake of oligopeptides. Thus, the oligopeptidase A was over-expressed in the macrophage model and under-expressed in the BALF model. Moreover, CsuC and CsuA were over-expressed in the host cell-free model (the BALF model) and under-expressed in macrophage model, because these proteins are involved in initial attachment to abiotic surfaces but not in the mechanism of adherence to biotic surfaces. Nevertheless, the explanation is not as obvious in the case of the modulation of DNA-directed RNA polymerase subunit beta’.

In the macrophage model, 3 predicted outer membrane proteins were over-expressed (a DcaP-like protein, a membrane protein and OmpA -iTRAQ ratio between 7.80 and 11.07). In the BALF model, 2 predicted outer membrane proteins were over-expressed (OmpW family protein and OmpA -iTRAQ ratio between 1.51 and 2.25). In the macrophage model, 21 predicted secreted proteins were also over-expressed (average iTRAQ ratio 7.88) and 10 were under-expressed (average iTRAQ ratio 0.25). In the BALF model, 17 predicted secreted proteins were over-expressed (average iTRAQ ratio 4.03) and 13 were under-expressed (average iTRAQ ratio 0.20). The modifications that *A. baumannii* undergoes as a result of host-induced stress include changes in membrane and secretome composition; these changes were greater in the macrophage model than in the BALF model (host cell-free model) probably due to pathogen-host cell interactions. We conclude that the outer membrane and secretome are changed by the host cells and host secretome.

Interestingly, we found that the UDP-N-acetylmuramate-L-alanine ligase (MurC) protein was strongly over-expressed in both *ex vivo* models (iTRAQ ratio of 99.08 in the BALF model, and iTRAQ ratio of 8.17 in macrophage model). We also observed that UDP-N-acetylglucosamine 1-carboxyvinyl transferase (MurA) was over-expressed in the BALF model, but not significantly over-expressed in the macrophage model (p > 0.05). In peptidoglycan synthesis, uridine-diphospho-*N*-acetylglucosamine is converted via MurA and MurB to UDP–*N*-acetylmuramic acid, to which alanine is added by MurC. These are essential steps in cell wall biosynthesis in bacteria [41, 42]. Moreover, MurA is required for full virulence in *Listeria monocytogenes* [43]. In this context, MurA has previously been suggested as a potential drug target, and effective inhibitors of this protein have been identified [44]. Detection of MurC polypeptides in humans could provide a method for diagnosis and/or prognosis of an infection. Levels of MurC polypeptides could be determined using assay techniques such as include antibody detection, ELISA assays, antibody sandwich assays and Western Blot analysis. Monoclonal antibodies against *A. baumannii* MurC similar to those generated by Zoeiby *et al.* could be used to detect MurC polypeptides. Zoeiby *et al.* used purified *P. aeruginosa* MurC protein to develop monoclonal antibodies against MurC. Moreover, Zoeiby *et al.* suggested that a specific inhibitor designed against *P. aeruginosa* MurC may display universal activity against all bacteria [45]. However, other authors have observed that C-1, a competitive inhibitor of ATP binding to the MurC enzyme, was equally effective at inhibiting MurC from *E. coli, Klebsiella pneumoniae and Proteus mirabilis*, but that MurC enzymes from more distantly related Gram-negative species such as *Acinetobacter baylyi, Pseudomonas aeruginosa and Haemophilus influenzae* were not inhibited [46]. More recently, MurC enzymes from *Escherichia coli and Pseudomonas aeruginosa* have been found to be inhibited by a novel class of pyrazolopyrimidines [47].

### Putative virulence-associated factors

Several putative virulence factors were modulated in both *ex vivo* models. Blast analysis (http://blast.ncbi.nlm.nih.gov/Blast.cgi) [48, 49] revealed significant alignment between the nucleotidyl transferase (AIS04894.1), which was found to be over-expressed in the macrophage model (but not significantly modulated (p > 0.05) in the BALF model) and UTP-glucose-1-phosphate uridylyltransferase (GalU) (query cover 100 % and Evalue = 0.0). The *GalU* gene encodes for production of UTP-glucose-1-phosphate uridyltransferase, an enzyme that catalyzes the formation of UDP-glucose and is known to have a key role in biosynthesis of cell-envelope-associated carbohydrates (*e.g.* lipopolysaccharides [LPS] and capsule) in a variety of bacterial species. In many Gram-negative pathogens, mutations in *GalU* lead to attenuated virulence, mainly because of changes in LPS or capsular structures. Formation of these polysaccharides is critical to bacterial virulence because this enables the bacteria to evade attack by the host immune system. The *GalU* gene has also been found to be important for pathogenesis of *e.g. Pseudomonas aeruginosa* and *Francisella tularensis* [50–54].

Bacteria and their hosts are involved in competition for available iron. In the host, iron is sequestered by ferritin, transferrin, haemoglobin, myoglobin, haptoglobin and haemopexin, thus decreasing the availability of free iron to concentrations below those needed for bacteria to persist in the environment. Therefore, bacteria must survive by competing with the host for iron through the expression of iron acquisition systems. These systems produce high affinity iron chelators known as siderophores, which are secreted into the extracellular milieu [55, 56]. Lipocalin-1 is secreted by the lingual glands, sweat glands, prostate, and secretory glands of the tracheobronchial tract, as well as by the nasal mucosa into BALF, sweat, saliva, tear, sputum and nasal fluids. Lipocalin-2, which is secreted by neutrophils, macrophages, dendritic cells and exocrine glands into trachea, lung, sputum, BAL, stomach, small intestine, pancreas, kidney, prostate, thymus and plasma, interferes with siderophore-mediated iron-uptake. Lipocalin-1 binds to different bacterial siderophores, including bacterial catecholate-type (enterobactin) and hydroxamate-type (ferricrocin) and mixed citrate-hydroxamates (aerobactin). Lipocalin-2 binds to different bacterial siderophores, including enterobactin-type bacterial siderophores and carboxy-mycobactins. To defend themselves against the host, some bacteria use countermeasures to subvert the iron-withholding effects of Lipocalin-2. For example, *Salmonella* and *Klebsiella* spp. glycosylate enterobactin, whereas *M. tuberculosis* modifies its carboxymycobactins. Both mechanisms sterically impair the ability of Lipocalin-2 to bind these siderophores [57–60]. NfuA-like has multiple functional roles related to iron-mediated stress. NfuA-like is involved in intracellular iron metabolism and, as demonstrated in an infection model, it also plays a role in the virulence of *A. baumannii* by protecting infecting bacteria from oxidative responses rather than providing iron to bacteria grown under iron—limited conditions imposed by cultured human alveolar epithelial cells and *G. mellonella* larvae [37]. In the present study, we observed that in both models, proteins involved in biosynthesis of the siderophore aerobactin were not significantly modulated (p > 0.05), probably because lipocalin-1 binds to aerobactin. We also observed that one of the under-expressed proteins (not significantly under-expressed (p > 0.05) in the BALF model) is associated with biosynthesis of the siderophore achromobactin (AIS06377.1). This may be because although achromobactin is an efficient hydroxycarboxylate siderophore at low pH (pH < 5), achromobactin could not compete with stronger siderophores such as catecholates at physiological pH [59]. In both models, NfuA-like was also over-expressed (although not significantly (p > 0.05), in the macrophage model). Altogether, these results suggest a third system for iron acquisition in *A. baumannii* strain AbH12O-A2, but which is not identified by current bioinformatic analysis. In a recent study, the structures of six novel siderophores termed fimsbactin A-F have been elucidated and the biosynthesis gene clusters for fimsbactin production have been identified in *A. baylyi* ADP1 and *A. baumannii* strains (ATCC 17978 and 6013150), although BLAST analysis showed that the fimsbactin gene cluster is not present in *A. baumannii* strain ABH12O-A2 (data not shown) [61].

In the macrophage model, we observed increased expression (not over-expressed in the BALF model: iTRAQ ratio, 1.26) of the Tig factor in the *ex vivo* samples. *De novo* protein folding in *Escherichia coli* is mainly orchestrated by the chaperone trigger factor and by DnaK and GroEL. The ribosome-bound trigger factor is the first chaperone to interact cotranslationally with nascent polypeptides. It has been suggested that a trigger factor homologue in *Streptococcus mutans* is a key regulator of stress tolerance, genetic competence and biofilm formation, all of which are critical virulence properties of this bacterium. In *Listeria monocytogenes*, a trigger factor homologue is involved in the stress response and associated pathogenicity [62–64].

We observed increased expression of the DnaK suppressor protein, which was significant (*p* < 0.05) in the BALF model, but not in the macrophage model. This protein plays an important role in the virulence of *Salmonella* and *Escherichia coli* [65].

In the BALF model, expression of the ClpX protein increased (but was not significantly over-expressed (*p* > 0.05) in the macrophage model). ClpX is required for virulence, biofilm formation and intracellular replication in *Staphylococcus aureus* [66].

Initial attachment to abiotic surfaces is the first step for colonization and subsequent biofilm formation on *e.g.* ventilator tubing and catheters. In *A.* baumannii 19606, the type I pili encoded by CsuA/BABCDE appears to be involved in this process [67]. In our host cell-free model (BALF model), the proteins OmpA (iTRAQ ratio = 1.51), CsuA and CsuC were over-expressed, suggesting biofilm activation by the host secretome that defends *A. baumannii* against host on medical devices. Another critical step in the pathogenesis of *A. baumannii* is the ability to adhere to eukaryotic cells, although the mechanisms of adherence are different for abiotic and biotic surfaces. It has been demonstrated that OmpA acts as a virulence factor in *A. baumannii* and has an important role in cell death through both mitochondrial and nuclear targeting [68]. The OmpA of *A. baumannii* 19606 also plays a partial role in biofilm formation on plastic, but is essential for attachment to biotic surfaces such as *C. albicans* and human alveolar epithelial cells. Interestingly, the absence of these cell appendages also favours bacterial attachment and invasion of epithelial cells. This may be due to greater exposure of other unknown bacterial adhesion and biofilm factors in the absence of pili [69]. In our macrophage model, OmpA (iTRAQ ratio = 7.80) was over-expressed and both CsuA and CsuC were under-expressed. Together these results suggest that contact between *A. baumannii* cells and macrophages may be an important factor determining underexpression of the CsuA/ BABCDE protein. Elucidation of the complex interactions between *A. baumannii*and both abiotic and biotic surfaces is important for a better understanding of the pathobiology of *A. baumannii* and also for identification of novel targets for the development of new antimicrobial strategies.

In the macrophage model, we observed increased expression of the PaaA protein in the *ex vivo* samples, but we did not observe significant (*p* > 0.05) repression of the PaaZ protein. In contrast, in the BALF model, we observed repression of PaaZ protein but no modulation (*p* > 0.05) of the PaaA protein. In the catabolism of phenylacetic acid by some bacteria, PaaA encodes part of the phenylacetic acid—coenzyme A ring hydroxylation system, and opening of the aromatic ring may be performed by PaaZ. In *Burkholderia cenocepacia, PaaA* insertional mutants were attenuated for virulence and interruption of increased virulence of *PaaZ* [70].

The success of a bacterial infection greatly depends on the ability of the bacteria to use external nutrients. Therefore, the proteolytic and lipolytic activities of extracellular proteins may play key roles in the establishment of *A. baumannii* infection [71]. In general, in the macrophage model, proteins with proteolytic activity were over-expressed, and in the BALF model, this type of protein was under-expressed. This may be due to continuous production of host proteins in the macrophage model but not in the BALF model as well as to over-expressed amino acid metabolism in the macrophage model and under-expressed amino acid metabolism in the BALF model.

We identified a cyclophilin (PPIase) (AIS06968.1), which was over-expressed in the macrophage model and not modulated in the BALF model. It has been established that PPIases may play a role during bacterial survival against macrophage attack, and it has been suggested that PPIases may act as virulence factors by interacting with some proteins from the host cell membrane, thus helping render the host cell more susceptible to penetration via conformational changes through cis-trans isomerization of peptidyl-prolyl bonds [72, 73]. These types of proteins have been reported to affect the phagocytosis of *Streptococcus pneumoniae* by macrophages [74].

In both models, the uncharacterized ABC transporter ATP-binding protein YjjK was over-expressed. The precise role of YjjK, encoding a putative ATP binding protein of an ABC transporter, is not yet known. However, YjjK is required for the entry and survival of *Porphyromonas gingivalis* in gingival epithelial cells [75]. It has also been suggested that YjjK is involved in *Francisella tularensis* virulence [76].

Eighteen genes have been identified in *A. baumannii* as being involved in growth on human ascites and shown to be essential for growth and survival during infection [77]. In the present study, we found 53 % of these essential proteins: 4 of these were over-expressed (OmpF, RpmA, CarA and PyrC), 4 were un-modulated (AroA, AroC, RstA and SecE) and AceE was under-expressed. The present findings are only partly consistent with those of the aforementioned study [77] as SpsC is not contained in the AbH12O-A2 genome and we did not detect8 of the proteins. However, these differences may be due to the undetected proteins occurring in amounts below the detection/identification level or to differences in the host microenvironment.

When *A. baumannii* infects the host airway, it is present in multiple locations, including in the interstitium between cells, embedded in mucus in the lumen, inside and adhering to macrophages and epithelial cells. Therefore, *A. baumannii* must adapt to multiple microenvironments and may express different proteins, depending on the microenvironment. We examined 2 *ex vivo* models of infection: a host cell-free model in which *A. baumannii* interacts with soluble host components present inside the infected lung, and an immune system cells model, in which *A. baumannii* interacts with macrophages. One limitation of the study is that we only simulated some conditions in the host airways and *A. baumannii* is present in many other microenvironments. Caution must therefore be used in extrapolating results of the present study to the human disease because *A. baumannii* populations that colonize the human airways are probably a mixture of *A. baumannii* from different microenvironments.

The AbH12O-A2 strain yielded over-expressed ‘hypothetical proteins’. Induced expression of hypothetical proteins strongly indicates that *A. baumannii* generates host resistance by unknown mechanisms. These genes are obvious candidates for subsequent functional characterization and for research aimed at determining their function within infection processes.

## Conclusions

This study is the first comprehensive overview of the *ex vivo* proteome of the multidrug resistant microbial pathogen *A. baumannii* and provides some insight into the potential role of putative virulence proteins *in vivo*. The *ex vivo* behaviour of *A. baumannii* was compared, for the first time, under different infection conditions—in BALF and in the presence of macrophages—as well as under *in vitro* conditions. A rapid enrichment technique was successfully used, together with MS/MS analysis, to characterize the *ex vivo* proteome of *A. baumannii* and showed that the proteome is significantly different from that of bacteria cultured *in vitro*. The changes in the proteome observed for the strain *A. baumannii* AbH12O-A2 indicate a response to stress resulting from interaction with host and modification of cell wall synthesis. We identified 2 over-expressed virulence-associated proteins with >15 peptides/protein in both *ex vivo* models (OmpA and an uncharacterized ABC transporter ATP-binding protein YjjK), which appear to be essential for pathogenesis and virulence in the airways. Overall, the data suggest that *A. baumannii* can use a variety of virulence mechanisms that enable it to adapt to and survive in vastly divergent environments. These data are helpful for elucidating the molecular mechanisms associated with the interaction between *A. baumannii* and host and represent an important step towards identification of diagnostic biomarkers, novel drug targets and potential vaccine candidates in the fight against pneumonia caused by *A. baumannii*.

## Methods

### Bacterial strain

A highly invasive multidrug-resistant (including carbapenems) *Acinetobacter baumannii* clone (AbH12O-A2), which infected more than 300 patients in the 12 de Octubre Hospital (Madrid, Spain) was used in this study.

The annotated sequence for AbH12O-A2 [GenBank CP009534.1] is available at the National Center for Biotechnology Information (NCBI) [78].

### Rat model of pneumonia and BALF (bronchoalveolar lavage fluid)

Male Wistar rats (INIBIC, A Coruña, Spain) of about 350 g weight were used in the experiment. Antibiotic-free pelleted food and water were provided *ad libitum* during the assay. The experiment was approved by the Ethics Committee for Animal Experimentation of A Coruna Hospital. The rats were anaesthetized intraperitoneally with sodium thiopental and were inoculated intratracheally under direct vision with a bacterial suspension, which was prepared as follows: bacteria were grown at 37 °C in LB until an optical density of 0.7 at 600 nm, washed with sterile saline solution and mixed 1:1 with a saline solution of porcine mucin at 10 % (wt/vol). The final inoculum was about 3 × 10^8^ CFUs/rat. At 21 h after inoculation, animals were euthanized by intraperitoneal injection of an overdose of sodium thiopental. The lungs were washed twice with about 45 ml normal saline (0.9 % NaCl). The concentrations of the original bacterial suspensions and of the BALF were determined by the plate count method. The BALF from three animals was centrifuged (3000 × g, 30 min at 4 °C) to remove cells, and the supernatant was filtered through a 0.22 μm membrane (low protein binding Millex-GP polyethersulfone membrane (Millipore, Bedford, U.S.A.)) to remove residual bacteria. Absence of viable bacteria was confirmed by culture on Mueller Hinton (MH) agar plates. BALF was stored at −70 °C for a maximum of 3 months until use.

### Histopathology

Paraformaldehyde-fixed lung tissue was embedded in paraffin, sectioned and stained with hematoxylin and eosin. Two to four sections from each lung of infected rats were examined.

### Incubation of *A. baumannii* in rat BALF

*Acinetobacter baumannii* strain AbH12O-A2 was streaked on MH nutrient agar. A single colony was then inoculated in 5 mL LB and grown overnight at 37 °C with vigorous shaking. 100 mL of LB (10 g/L tryptone + 5 g/L yeast extract dissolved in saline solution instead of water) and 100 ml of modified LB (40 g/L tryptone + 20 g/L yeast extract dissolved in saline solution instead of water + 75 % (v/v) BALF as a physiologically relevant source of host proteins to simulate conditions in the respiratory tract) were inoculated with a 1:100 dilution of the overnight culture and grown simultaneously for 21 h at 37 °C and 5 % CO_2_ without shaking. Three independent biological replicates of each culture were prepared but only 2 were used for the ITRAQ. Cells (OD600 nm = 1.0-1.4) were harvested by centrifugation at 2800 × g for 20 minutes at 4 °C, washed twice by suspending in cold 0.9 % NaCl and centrifuged again under the same conditions. The pellets were frozen and stored at −70 °C until needed.

### Macrophage infection with *A. baumannii*

*Acinetobacter baumannii* strain AbH12O-A2 was streaked on MH nutrient agar. A single colony was then inoculated in 5 mL LB and grown overnight at 37 °C with vigorous shaking. The overnight culture was harvested, washed once and resuspended in tissue culture medium: DMEM with glucose and L-glutamine (4.5 g/L dextrose + 3.7 g/L NaHCO_3_ + 0.015 g/L phenol red + 0.110 g/L sodium pyruvate + 0.200 g/L CaCl_2_ + 0.100 mg/L Fe(NO_3_)_3_.9H_2_O + 0.098 g/L MgSO_4_ + 0.400 g/L KCl + 6.400 g/L NaCl + 0.007 g/L i-Inositol + 0.109 g/L NaH_2_PO_4_ + 15 amino acids and vitamins (BioWhittaker Lonza, Verviers, Belgium)) supplemented with 10 % fetal bovine serum and 1 % penicillin/streptomycin. The concentration of bacterial cells was then determined.

To mimic host microbe interactions involving phagocytic immune system cells the RAW 264.7 macrophage-like cell line was plated in cell culture flasks at 3 × 10^7^ cells/flask and incubated overnight at 37 °C with 5 % CO2. Macrophage cells were washed twice with 20 ml/flask of 0.9 % NaCl. The tissue culture medium was then added to each flask, and 1 mL of the *A. baumannii* in the tissue culture medium was added to each flask with a multiplicity of infection of 3 (i.e. 3 times more bacterial cells than macrophages were used). To increase the uptake of *A. baumannii* and synchronize infection, flasks were centrifuged at 250 × g for 5 min at room temperature and then incubated at 37 °C with 5 % CO_2_ for 21 h without shaking. Three independent biological replicates of each culture were prepared but only 2 were used for the ITRAQ. The sample was harvested 21 h post infection. Extracellular bacteria were isolated from infected macrophage cultures. To isolate extracellular bacteria, infected macrophage cultures were washed three times with Hank’s buffered salt solution to remove any bacteria that were not inside adherent cells. The harvested sample and the wash solutions were centrifuged (500 × g for 2 min at 4 °C to pellet any nonadherent macrophage cells) and the supernatants were combined. The supernatant was centrifuged (1500 × *g* for 20 min at 4 °C) to pellet the bacteria. The pellet was resuspended in phosphate-buffered saline (PBS) and filtered through a Transwell filter membrane system (3.0-μm pore size; BD Falcon, Erembodegem, Belgium). Macrophage cells were very scarce. Bacteria then were washed twice by suspending them in cold PBS and centrifuging (2800 × g for 20 minutes at 4 °C). The pellets were frozen and stored at −70 °C until needed. Control *A. baumannii* cells were collected, washed and treated in the same way as above but in the absence of macrophages.

### Protein extraction

The resultant pellet was resuspended in disintegration buffer (7.8 g/L NaH_2_PO_4_, 7.1 g/L Na_2_HPO_4_, 0.247 g/L MgSO_4_.7H_2_O) + protease inhibitor mix (GE Healthcare, Piscataway, USA) + nuclease mix (GE Healthcare, Piscataway, USA) and sonicated on ice for 3 periods of 5 min. The unbroken cells were separated by centrifugation at 1500 × *g* at 4 °C. The supernatant was centrifuged for at least 30 min at 4 °C and 4500 × g before being clarified through a 0.22 μm membrane (low protein binding Millex-GP polyethersulfone membrane from Millipore, Bedford, U.S.A.) to remove the cell debris. Finally, the extract was processed with a 2-DE Cleanup Kit (GE Healthcare, Piscataway, USA), following the manufacturer’s instructions. The concentration of protein was measured using the Bio-Rad protein assay (Bio-Rad, Munich, Germany).

### iTRAQ labelling and LC-MALDI/TOF analysis

Labelling, 2-D liquid chromatography and MALDI-TOF/TOF MS analysis of the samples were performed as previously described [79], and stage tips were used for peptide desalting. Briefly, 30 μg of protein from each condition was reduced, cysteine was blocked, digested with trypsin and labelled with respective isobaric tags using iTRAQ reagent Multiplex kit (AB Sciex Ltd., Foster City, CA), according to the manufacturer’s protocol. The sample labelling was as follows: iTRAQ tags 114: control BALF model; iTRAQ tags 115: sample BALF model; iTRAQ tags 116: control macrophage model; iTRAQ tags 117: sample macrophage model. In the first dimension, peptides were fractionated by basic reversed-phase chromatography in a 1200 HPLC system (Agilent) and were collected using a Gilson FC203B fraction collector (Gilson, Middleton, WI). The collected peptide fractions were desalted with the aid of home made stage tips and separated by reversed-phase chromatography at acid pH in a nanoLC system (Tempo, ABSciex, Foster City, CA). The peptides were desalted and concentrated in a trapping column (0.5 × 2 mm, Michrom Bioresources, Auburn, CA) at a flow rate of 15 μL/min for 15 min and loaded onto a C18 column (Integrafit C18, Proteopep II, 75 μm i.d., 15 cm, 5 μm, 300 Å; New Objective, Woburn, MA). Peptides were eluted at a flow rate of 0.35 μL/min during a 2 h linear gradient from 2 to 40 % B (mobile phase A: 0.1 % trifluoroacetic acid, 2 % acetonitrile; mobile phase B: 0.1 % trifluoroacetic acid, 95 % acetonitrile), mixed with α-cyano matrix (4 mg/mL at a flow rate of 1.2 μL/ min), automatically deposited on a MALDI plate using a MALDI spotter (SunCollect, Sunchrome, Friedrichsdorf, Germany) and were finally analyzed in a 4800 TOF/TOF system (ABSciex, Foster City, CA). The chromatograms were composed by 480 spots, each comprising a 15 s deposition. Explorer v.4.2 (ABSciex, Foster City, CA) software (series 4000) was used to generate both the spectra and peak list. Plate model and default calibration of the MALDI plate were performed at a laser voltage of 3400 kV and 1000 shots/spectrum. Samples were automatically analyzed in MS mode at a laser voltage of 3800 kV and 1500 shots/spectrum.

Automated precursor selection was carried out by a Jobwide interpretation method, which selects up to 12 precursors per fraction. The lower threshold of signal to noise (50) and trypsin autolytic peptides and other background ions were excluded. The laser voltage was 4800 kV, and 2000 shots/spectrum were acquired using a medium-range CID collision energy.

LC-MALDI-TOF/TOF data were analyzed using Protein Pilot 4.0 software (ABSciex, Foster City, CA) as the search engine for protein identification. Protein Pilot search parameters were as follows: Cys-alkylation, iodoacetamide; iTRAQ 4plex quantitation mode, biological modifications; digestion with trypsin; All searches were performed against the non-redundant NCBI library (http://ncbi.nlm.nih.gov) database comprising annotated proteins of *Acinetobacter baumannii* AbH12O-A2 and pMMA2 plasmid. The search effort comprised thorough ID and detection protein threshold of unused ProtScore (Conf > 1.3 (95.0 %)). The scoring model was defined by the Paragon algorithm. Bias and background correction were applied to correct experimental differences in total amount of protein used in the experiment and label interference, respectively. Proteins showing sample/control protein expression ratios below 0.5 or above 1.5 (p < 0.05) were considered to be respectively under- or over-expressed and were selected for further analysis.

### Purity of the starting material

The MS analysis of *A. baumannii* in BALF model and in macrophage model did not reveal any of the most abundant BALF proteins (serum albumin, IgG, IgA, transferrin, haptoglobin and antitrypsin) [80], nor the most abundant protein component of alveolar surfactant (surfactant protein A) [81], nor the very abundant enzymes phosphoglycerate kinase (cytosol of macrophages) and metallopeptidase 9 (macrophage membranes) [82]. We therefore, concluded that the starting material for the *A. baumannii* proteome analysis was not (or only minimally) contaminated with host material. This was not surprising because BALF proteins sediment at much higher centrifugation speeds than bacteria and because we could barely see nonadherent macrophage cells (see section “Macrophage infection with *A. baumannii*”).

### Bioinformatics

Protein function was determined by http://www.ncbi.nlm.nih.gov.

HHomp, http://toolkit.tuebingen.mpg.de/hhomp# was used for the prediction of outer membrane proteins.

The presence of export signals was predicted by use of a combination of algorithms (SignalP 4.1 (http://www.cbs.dtu.dk/services/SignalP/), Phobius (http://phobius.sbc.su.se/), PrediSi (http://www.predisi.de/), TatP 1.0 (www.cbs.dtu.dk/services/TatP-1.0), Tatfind 1.4 (http://signalfind.org/tatfind.html), SecretomeP 2.0 (www.cbs.dtu.dk/services/SecretomeP), TMHMM (http://www.cbs.dtu.dk/services/TMHMM/), DAS-TMfilter (http://www.enzim.hu/DAS/DAS.html), LipoP 1.0 (www.cbs.dtu.dk/services/LipoP), DOLOP (http://www.mrc-lmb.cam.ac.uk/genomes/dolop/), and LIPO (http://services.cbu.uib.no/tools/lipo)). [see Additional file 3].

### RNA extraction and RT-PCR

Total RNA was extracted using the High Pure RNA isolation kit (Roche, Mannheim, Germany), according to the manufacturer’s instructions. PCR without reverse transcriptase confirmed the absence of DNA. Templates of 100 ng of total RNA were used in the target gene studies. Real-time PCR analysis of gene expression was performed in duplicate with specific internal oligonucleotide primers and the TaqMan probe (Universal ProbeLibrary-UPL, Roche, Mannheim, Germany). All primers and UPL probes used in the RT-PCR study are shown in Additional file 4.

### Availability of supporting data

The proteomics data sets supporting the results of this article are included within the article and its Additional file 5.

## Additional files

Additional file 1:
**Workflow of the proteomic experiment.** Strategy used to recover and identify the proteins of *A. baumannii* in 2 ex vivo models for proteomic analysis. Additional file 2:
**Histopathology shows signs of consolidated pneumonia in infected animals.** A), C) and E) representative low (×4) - and B), D) and F) high (×40)-power histological sections of lungs from three rats infected with *A. baumannii* for 21 h. A – F haematoxylin and eosin staining. Additional file 3:
**Prediction of exoproteins. This figure indicates the sequential use of different algorithms into a majority vote decision.** Coding sequences were scanned for the presence of signal peptide specific to Sec pathway and Tat pathway. Coding sequences exhibiting no signal peptide were screened as potential nonclassically secreted proteins using SecretomeP 2.0. Proteins predicted as secreted were then asked for the presence of cell-envelope retention domain and erased from the output in positive case. Y, yes; N, no. Additional file 4:
**RT-PCR analysis of different genes. Primers and Univesal ProbeLibrary (UPL, Roche) probes used in this study.**
Additional file 5:
**Protein identification data.** Excel workbook containing 2 worksheets with the complete report for the proteins in both models exported from Protein Pilot software.

## Abbreviations

BALF: Bronchoalveolar lavage fluid
LB: Luria-Bertani broth
LPS: Lipopolysaccharides
NCBI: National Center for Biotechnology Information
MH: Mueller Hinton
DMEM: Dulbecco’s Modified Eagle’s Medium
PBS: Phosphate-buffered saline
