# Supplementary material for: Quantitative proteomic analysis of host—pathogen interactions: a study of Acinetobacter baumannii responses to host airways
Source: BMC Genomics. 2015 May 30;16(1):422. doi: 10.1186/s12864-015-1608-z (PMC4449591; doi:10.1186/s12864-015-1608-z)
Supplement: Additional file 1: — Workflow of the proteomic experiment. Strategy used to recover and identify the proteins of A. baumannii in 2 ex vivo models for proteomic analysis. [file 12864_2015_1608_MOESM1_ESM.pptx]

## Slide 1
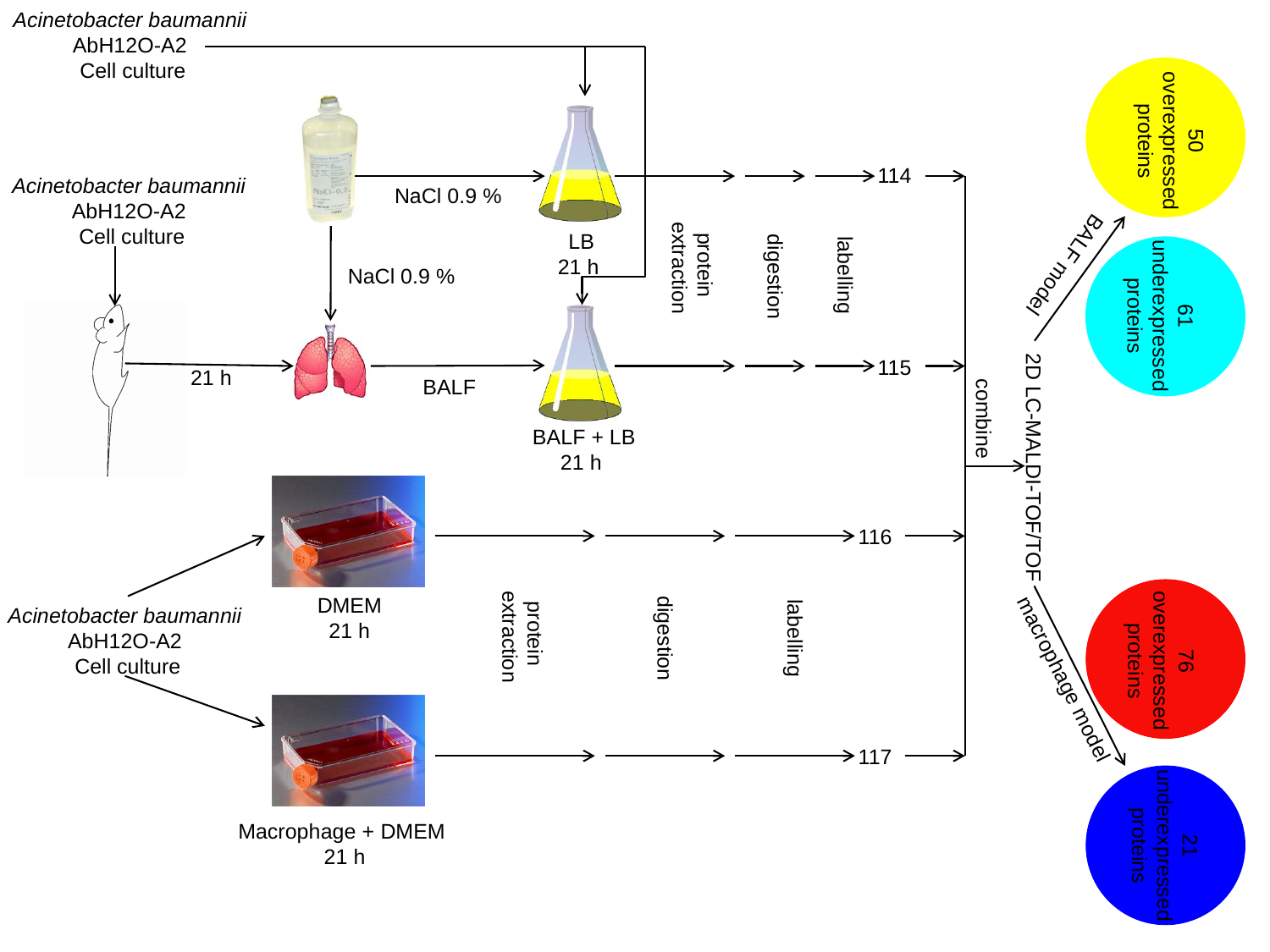

Acinetobacter baumannii
AbH12O-A2
Cell culture
50 overexpressed proteins
NaCl 0.9 %
LB
21 h
NaCl 0.9 %
21 h
BALF
114
Acinetobacter baumannii
AbH12O-A2
Cell culture
61
underexpressed
proteins
protein
extraction
BALF model
labelling
digestion
115
combine
BALF + LB
21 h
2D LC-MALDI-TOF/TOF
116
76
overexpressed proteins
DMEM
21 h
Acinetobacter baumannii
AbH12O-A2
Cell culture
protein
extraction
digestion
labelling
macrophage model
117
21
underexpressed
proteins
Macrophage + DMEM
21 h
