# Supplementary figures and images for: Quantitative proteomic analysis of host—pathogen interactions: a study of Acinetobacter baumannii responses to host airways
Source: BMC Genomics. 2015 May 30;16(1):422. doi: 10.1186/s12864-015-1608-z (PMC4449591; doi:10.1186/s12864-015-1608-z)

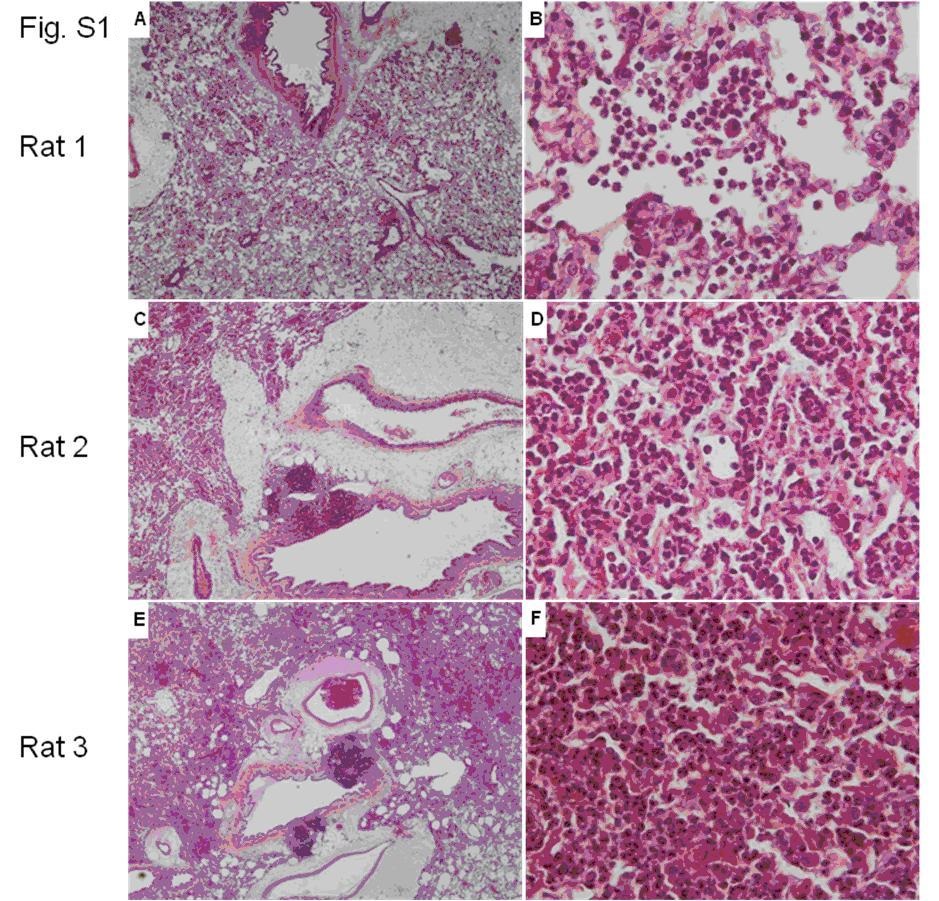

Supplement: Additional file 2: — Histopathology shows signs of consolidated pneumonia in infected animals. A), C) and E) representative low (×4) - and B), D) and F) high (×40)-power histological sections of lungs from three rats infected with A. baumannii for 21 h. A – F haematoxylin and eosin staining. [file 12864_2015_1608_MOESM2_ESM.jpeg]

## Slide 1
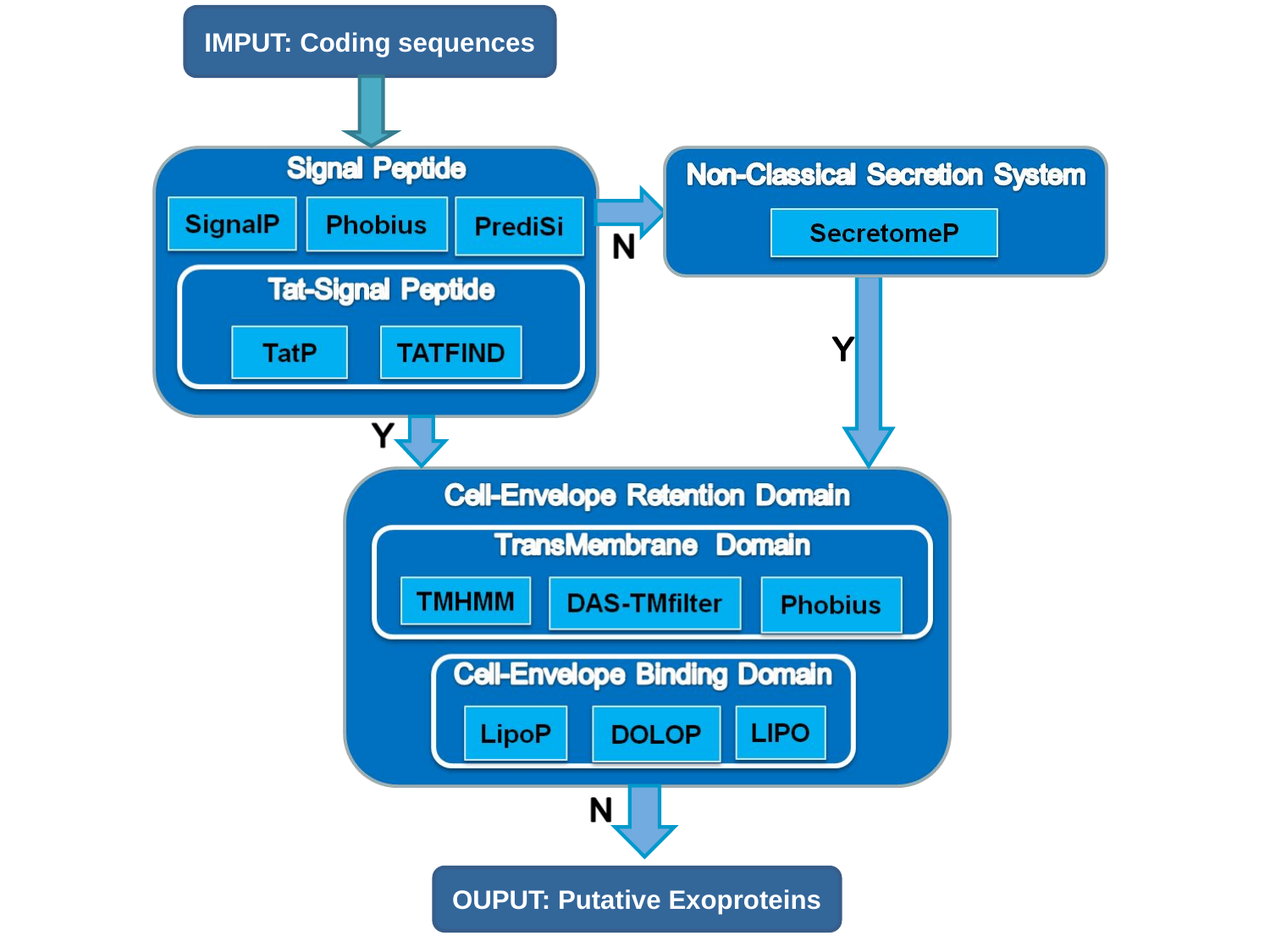

IMPUT: Coding sequences
OUPUT: Putative Exoproteins

Supplement: Additional file 3: — Prediction of exoproteins. This figure indicates the sequential use of different algorithms into a majority vote decision. Coding sequences were scanned for the presence of signal peptide specific to Sec pathway and Tat pathway. Coding sequences exhibiting no signal peptide were screened as potential nonclassically secreted proteins using SecretomeP 2.0. Proteins predicted as secreted were then asked for the presence of cell-envelope retention domain and erased from the output in positive case. Y, yes; N, no. [file 12864_2015_1608_MOESM3_ESM.ppt]
