# Supplementary material for: Quantitative proteomic analysis of host—pathogen interactions: a study of Acinetobacter baumannii responses to host airways
Source: BMC Genomics. 2015 May 30;16(1):422. doi: 10.1186/s12864-015-1608-z (PMC4449591; doi:10.1186/s12864-015-1608-z)
Supplement: Additional file 4: — RT-PCR analysis of different genes. Primers and Univesal ProbeLibrary (UPL, Roche) probes used in this study. [file 12864_2015_1608_MOESM4_ESM.docx]

Additional file 4. Primers and Univesal ProbeLibrary (UPL, Roche) probes used in this study.

| Gene | Locus tag | Forward primer | Reverse primer | UPL |
| --- | --- | --- | --- | --- |
| *DNA repair* | AbH12O-A2-0172 | tttttattgcagctttacgttcac | ttattccaatacttcgctttgtttt | 89 |
| *ClpX* | AbH12O-A2-0473 | caagctttgcttaagatgattgaa | gtggatgcttacgtccacct | 31 |
| *NfuA* | AbH12O-A2-0942 | Gacgcggttattgactacaaca | agagtttggagcacggaaag | 119 |
| *PaaA* | AbH12O-A2-1261 | Agatgtcgctgcaattggtt | aagcgcaacttggttcaca | 43 |
| *PpiA* | AbH12O-A2-2202 | Cgcgttatcgacggtttc | gcatcacgagttgctttttct | 77 |
| *CsuC* | AbH12O-A2-2305 | accattgcatcggtcttacc | agccaatgaaaaggcaactg | 139 |
| *OmpA* | AbH12O-A2-2965 | ttggttcaactggagcaactt | cgtagttcttggtggtcacttg | 40 |
| *YjjK* | AbH12O-A2-3297 | tttgcaagcgcatcaaaat | gcgtcttgatcaggtatttgc | 115 |
| *OmpW* | AbH12O-A2-0294 | gctggccaagttggttttaat | ctgcataacgcacatctacaaat | 62 |
